# Stillbirth Discourse on Instagram and X (Formerly Twitter): Content Analysis

**DOI:** 10.2196/73980

**Published:** 2025-09-24

**Authors:** Abigail Paradise Vit, Daniel Fraidin, Yaniv S Ovadia

**Affiliations:** 1 Information Systems, The Max Stern Yezreel Valley College, D.N. Emek Yezreel, Kibbutz Jezreel, Emek Yezreel, 1930600, Israel, +972 509903930; 2 Department of Obstetrics and Gynecology Division, Barzilai University Medical Center, Ashkelon, Israel

**Keywords:** emotion analysis, Instagram, misinformation, stillbirth, topic modeling, X (formerly Twitter)

## Abstract

**Background:**

Stillbirth, the loss of a fetus after the 20th week of pregnancy, affects about 1 in 160 deliveries in the United States and nearly 1 in 70 globally. It profoundly affects parents, often resulting in grief, depression, anxiety, and posttraumatic stress disorder, exacerbated by societal stigma and a lack of public awareness. However, no comprehensive analysis has explored social media discussions of stillbirth.

**Objective:**

This study aimed to analyze stillbirth-related content on Instagram and X (formerly Twitter) by (1) identifying dominant themes using topic modeling, evaluated using latent Dirichlet allocation, non-negative matrix factorization (NMF), and BERTopic; (2) detecting influential hashtags via co-occurrence network analysis; (3) examining sentiments and emotions using transformer-based models; (4) categorizing visual representations of stillbirth on Instagram (Meta) through manual image analysis with a predefined codebook; and (5) screening for misinformation relating to stillbirth on X.

**Methods:**

Stillbirth-related posts were collected via RapidAPI (N=27,395), with Instagram posts (#stillbirth: n=7415; #stillbirthawareness: n=8312; 2023‐2024) and X posts (#stillbirth: n=11,668; 2020‐2024) analyzed using Python 3.12.7 (Python Software Foundation), with NetworkX for hashtag co-occurrence networks and the PageRank algorithm; comparative analyses were restricted to 2023‐2024 due to Instagram application programming interface constraints. Topic modeling was evaluated using latent Dirichlet allocation, NMF, and BERTopic, with coherence scores guiding our model selection. Sentiment and emotion were analyzed using transformer-based RoBERTa and DistilRoBERTa. Misinformation screening was applied to X posts. On Instagram, 2 representative image samples (n=366) were manually categorized using a predefined codebook, with the interrater reliability being assessed using Cohen Kappa.

**Results:**

Health-related hashtags (eg, #COVID19) appeared more frequently on X. Topic modeling showed that NMF achieved the highest coherence scores (#stillbirthawareness=0.624 and #stillbirth=0.846 on Instagram, #stillbirth=0.816 on X). Medical misinformation appeared in 27.8% (149/536) of tweets linking COVID-19 vaccines to stillbirth. In the image analysis, “Image of text” was most common, followed by remembrance visuals (eg, gravesites and stillborn infants). The interrater reliability was strong, κ=0.837 (95% CI 0.773‐0.891) and κ=0.821 (95% CI 0.755‐0.879), with high Pearson correlation (*r*=0.999; *P*<.001) and no significant difference (*χ*²_7_=12.4; *P*=.09). The sentiment analysis found that positive sentiments exceeded negative sentiments. The emotion analysis showed that fear and sadness were dominant, with fear being more prevalent on X.

**Conclusions:**

Instagram emphasizes emotional expression while X focuses on public health and informational content. Evidence-based communication is necessary to counter misinformation, especially on X, whose real-time affordances amplify fear-based narratives during crises, such as COVID-19. In addition, Instagram’s visual and commemorative content offers an opportunity to legitimize parental grief and to validate and humanize loss by directly involving bereaved parents in awareness campaigns. Platform-specific strategies and stronger moderation could enhance health discourse credibility. Future research should examine targeted approaches to counter misinformation and assist affected populations.

## Introduction

### Background

Stillbirth is defined as the delivery of a fetus that shows no signs of life at or after the 20th week of pregnancy [1]. Despite advances in medicine, about 2 million stillbirths occur every year worldwide [2]. Stillbirth occurs in approximately 1 in 160 deliveries in the United States [1] and nearly 1 in 70 globally [2]. Stillbirths may be caused by various factors, including placental abnormalities; infections; maternal health conditions, such as obesity, poorly controlled gestational diabetes mellitus, preeclampsia, and severe iron deficiency anemia; and inadequate prenatal care [1-4].

Experiencing a stillbirth can be an emotionally devastating event for parents, profoundly affecting their psychological well-being [5,6]. Studies have shown that parents who have experienced stillbirth are significantly more likely to experience a range of emotional and psychological challenges than those who have experienced a live birth. These challenges include symptoms of stress, depression, anxiety, social phobia, agoraphobia, anger, negative cognitive appraisals, such as feelings of failure and long-term guilt [7,8], and, in many cases, posttraumatic stress disorder [9-11].

After experiencing a stillbirth, parents face a deeply challenging and complex grief process [8,12], further exacerbated by several factors. One major issue is the lack of public awareness about stillbirth. The term itself is not widely known or understood, nor is its significant emotional impact on parents [13-15]. Another challenge is that stillbirth loss may not be recognized as a real death. Many stillborn babies are not formally named or buried, and parents may be unaware of the exact location of their child’s burial [16-18]. This lack of recognition can complicate the grieving process, leaving parents feeling that their loss has been minimized or dismissed. Furthermore, the parents’ grief can be amplified by societal stigma and blame, especially in cultures where stillbirth is taboo or reproduction is considered central to a woman’s identity. In such settings, stillbirth may be unfairly viewed as a personal failure, adding an additional emotional burden to an already painful experience [18]. Compounding this, societal attitudes may fail to recognize the significance of the loss and grant it legitimacy, with some expecting parents to quickly continue with their lives and have another child [19].

Those perceptions leave parents feeling isolated, with disconnection from their social environment further deepening their sense of marginalization and loss of support [5]. A profound sense of isolation underscores the importance of supportive networks. Research indicates that women who experience strong family support report significantly lower levels of anxiety and depression than those who lack such support [20]. In addition, support groups have been found to significantly reduce posttraumatic stress responses in women who have experienced stillbirth [20].

### Theoretical Framework

Continuing bonds theory posits that bereaved individuals maintain ongoing connections with the deceased, reframing grief as an adaptive process of sustaining attachments [21,22]. In digital spaces, this may manifest through digital memorialization and emotional expression, where social media enables shared narratives and visuals to preserve the deceased’s presence [23]. For stillbirth grief, often disenfranchised, platforms like Instagram facilitate visual tributes that embody continuing bonds theory, while X (formerly Twitter) foregrounds public health discourse and supports spaces where grief is articulated and shared [24]. This study applies continuing bonds theory to analyze how these digital practices mitigate grief [21].

As part of the grieving process, bereaved parents navigating stillbirth confront a unique, often unseen form of loss [25]. Social media provides a space for digital memorialization, enabling ongoing connection with their baby’s memory through shared narratives and visuals [21,26]. Grounded in continuing bonds theory, we hypothesize that Instagram’s visual affordances and X’s real-time discourse facilitate support and health discourses. Despite the presence of stillbirth content across digital platforms, existing studies are limited in scope. This study addresses this gap by systematically analyzing stillbirth content on social media, exploring themes, emotions, sentiments, misinformation, and visual representations.

### Related Work

In this section, we review prior studies that have analyzed social media content relating to miscarriage, abortion, and stillbirth. In addition, we examine research relating to the spread of medical misinformation on social media platforms.

#### Miscarriage, Abortion, and Stillbirth on Social Media

Several studies have explored the representation of miscarriage and abortion in social media discourse. Miscarriage is defined as the spontaneous loss of a pregnancy before 20 weeks, while abortion refers to a medical procedure that terminates a pregnancy [27]. Cesare et al [28] conducted a large-scale analysis of 291,443 tweets posted between 2017 and 2018, focusing on miscarriage and preterm birth. Using latent Dirichlet allocation (LDA), the study identified thematic patterns within the textual data; however, it was limited to a single platform (X), excluded stillbirth-related content, and did not incorporate sentiment or emotional analysis. Similarly, Philippe et al [29] examined tweets on X to assess public opinions and sentiment surrounding miscarriage and abortion. Their study concentrated on engagement metrics but did not include stillbirth in its scope of analysis.

Additional studies addressing pregnancy loss on social media have primarily focused on miscarriage and been based on limited, small sample sizes. For instance, Mercier et al [30] analyzed 200 Instagram posts tagged with #ihadamiscarriage. Similarly, Callen and Oxlad [31] used content analysis to examine 270 posts and 3484 comments within a closed Facebook (Meta) support group dedicated to miscarriage.

Stillbirth is clinically distinct from miscarriage, as it occurs at or after 20 weeks of gestation and typically involves full labor—and in some cases, may even require a cesarean section [32,33]. This combination of physical demands and the psychological trauma of delivering a nonviable infant makes stillbirth a uniquely complex and emotionally challenging experience. However, despite its profound impact, stillbirth remains significantly underrepresented in social media research. The limited number of studies that have directly examined stillbirth-related content on social media is narrow in both design and scope, often lacking in methodological depth, multimodal analysis, or large-scale data coverage. For example, Sani et al [34] and Geusens et al [35] analyzed small samples of YouTube videos (50 and 19, respectively) using qualitative methods to explore themes, such as grief, rituals, and parental experiences. Chan et al [36] assessed the reach of a public health campaign (“Still Six Lives”) through social media metrics and surveys. However, these studies did not use computational methods, lacked sentiment and emotion analyses, and were restricted to a single platform.

This study seeks to address this gap by presenting the first large-scale, cross-platform, and multimethod analysis of stillbirth-related discourse on social media, focusing on X and Instagram. These 2 platforms are more suited for stillbirth content analysis than other platforms, such as Facebook. Instagram’s visual focus enables a thorough analysis of grief and remembrance through images [30]. X’s real-time, public nature facilitates the tracking of emotional expressions and themes [37]. Facebook’s more private, network-based structure generally yields less accessible and diverse public discourse on such sensitive topics than the platforms of Instagram and X [38]. Specifically, this study integrates topic modeling, sentiment and emotion detection, identification of medical misinformation, hashtag network analysis, temporal trend analysis, and manual coding of visual content across both platforms. Together, these analyses provide a more comprehensive understanding of how stillbirth is discussed, visualized, and represented within the digital public sphere.

#### Misinformation on Social Media

Medical misinformation on social media has been widely explored in recent research [39,40]. For instance, several studies have used automated misinformation detection techniques, including the use of deep learning models, to identify and classify false or misleading health-related content across various platforms [41,42].

During the COVID-19 pandemic, the prevalence and impact of health-related misinformation increased dramatically, particularly regarding vaccines. Of concern, a meta-analysis among vaccinated individuals found no safety concerns for COVID-19 vaccination during pregnancy [43]. Moreover, a systematic review and meta-analysis found that COVID-19 vaccination was associated with a lower risk of stillbirth [44]. Nevertheless, claims linking COVID-19 vaccines to infertility, miscarriage, and stillbirth without any basis in scientific evidence were circulated widely on platforms, such as Facebook, Instagram, TikTok, and X, shaping public discourse and fueling vaccine hesitancy [45]. This prompted a surge of research focusing on the dynamics and consequences of vaccine-related misinformation in digital environments. However, studies that specifically examine the intersection of stillbirth and misinformation on social media remain limited.

## Methods

### Software

All data analyses and visualizations were performed using Python version 3.12.7 (Python Software Foundation).

### Data Collection

Posts were collected from the social media platforms Instagram and X, both of which were purposefully selected for their distinct roles in shaping social media discourse. Instagram, as a visually driven platform, facilitates personal storytelling and emotional expression, primarily through images and stories [46,47]. This makes it particularly well-suited for capturing the visual and affective dimensions of stillbirth experiences. In contrast, X is a predominantly text-based medium that functions as a dynamic arena for public debate [48] and the rapid dissemination of health-related information [49]. Together, these platforms support a comprehensive exploration of the diverse narratives, emotional expressions, and visual practices of stillbirth discourse in the digital public sphere.

The data for this study were obtained using the RapidAPI platform, which provides access to social media content through an application programming interface (API). We collected all available data provided by the API. For Instagram, we retrieved posts containing the 2 most used hashtags relating to stillbirth: #stillbirth and #stillbirthawareness. These posts were collected between January 1, 2023, and February 20, 2024, and included the publication date, accompanying text, and associated image. For X, we collected all available posts associated with the hashtag #stillbirth. X data were collected between January 1, 2020, and December 31, 2024, and included the tweet ID, publication date, and text.

We acknowledge the temporal mismatch between the 2 datasets. While the X data spans 5 years (2020‐2024), Instagram data is limited to the 2023‐2024 period. This discrepancy results from technical limitations in Instagram’s API at the time of data collection in 2024, which restricted our access to recent content only. This divergence introduces a potential methodological limitation, particularly for comparative analyses. To address this, any cross-platform comparisons were limited to the overlapping time frame of 2023‐2024. However, in analyses that focused solely on X, the full dataset (2020‐2024) was used to provide a broader temporal context.

### Hashtag Co-Occurrence Network

As mentioned in the “Data Collection” section, our dataset comprised 3 subsets of data based on posts containing the hashtags #stillbirth (on both X and Instagram, forming 2 subsets) and #stillbirthawareness (1 subset on Instagram). Since individual posts often included additional hashtags beyond those used for initial retrieval, we conducted a hashtag co-occurrence network analysis to explore how hashtags clustered and co-occurred within posts.

Two hashtag co-occurrence networks were constructed: one based on posts containing #stillbirth on X, and the other from Instagram posts featuring the same hashtag. To ensure comparability between platforms, the analysis was restricted to a consistent time frame spanning the years 2023‐2024.

In each network, each node represents a hashtag, and an edge connects 2 hashtags if they appear together in the same post. The network is undirected, and the weight of each edge is determined by the number of posts in which hashtags co-occurred.

For each network, PageRank scores were calculated to identify the most influential hashtags. PageRank is an algorithm developed by Google’s founders to rank web pages based on their importance within a network [50]. It assigns higher scores to nodes that are connected to several other important nodes. The PageRank algorithm is widely used in network analysis as a centrality measure [51].

For a hashtag’s co-occurrence network, PageRank helps identify the most prominent hashtags by analyzing their connections, where the importance of a hashtag is determined based on its frequent co-occurrence with other hashtags. For each network, we analyzed the nodes with the highest PageRank scores in order to identify the most influential hashtags within the discourse. This centrality-based approach provided deeper insights into the hashtags shaping discussions on social media about stillbirth.

The PageRank calculation was performed with its default parameters using the NetworkX library in Python. To enable a meaningful comparison of the prominence of hashtags across Instagram and X, we addressed the challenge that PageRank scores are inherently platform-specific due to differences in network structures and co-occurrence patterns [52]. As such, a direct comparison of absolute PageRank values across platforms may be misleading. To overcome this limitation, we used 2 complementary strategies: First, we ranked all hashtags within each platform according to their PageRank scores. This approach allowed us to identify the most influential hashtags within each network based on their internal structure and relative centrality.

Second, we calculated a normalized relative weight for each hashtag. This was defined as the sum of the hashtag’s edge weights across all co-occurrence connections, divided by the total sum of edge weights in the respective platform’s network. This normalization enabled a more reliable and consistent comparison of different hashtags’ prominence across platforms.

It is important to note that it was not possible to statistically assess whether the difference in relative weight for each individual hashtag across platforms was significant, as each hashtag is represented by a single data point per platform. In the absence of multiple observations per hashtag, standard significance testing could not be applied.

Finally, to visually explore the hashtag co-occurrence structure, we constructed network graphs for both platforms (X and Instagram) using Cosmograph (Nikita Rokotyan) [53]. Cosmograph is a browser-based tool designed for the visualization of large-scale network graphs and machine learning embeddings. In this study, we used the tool with its default settings. In each network, hashtags are shown as nodes, with size, color, and label color reflecting PageRank centrality, and edges weighted by hashtag co-occurrence frequency.

### Topic Modeling

To explore the dominant narratives surrounding stillbirth on social media, we applied topic modeling to categorize posts into distinct thematic clusters. This analysis was conducted separately for 3 subsets. Of these, 2 subsets of posts containing the hashtag #stillbirth on both X and Instagram, and 1 subset of posts that were tagged with #stillbirthawareness on Instagram. All hashtags were removed from the input prior to modeling. This decision was made to avoid redundancy, as hashtags were already analyzed in the hashtag co-occurrence networks. By focusing exclusively on the textual body of the posts, the topic modeling aimed to capture the narrative content, without any influence from metadata or tag-based categorization.

Since the goal of this analysis was to identify prevalent topics on social media in the context of stillbirths, we did not restrict the datasets to the same time period across platforms. Instead, the analysis was conducted using the full range of available data from each platform. As such, the results should not be interpreted as a direct comparison between platforms.

Prior to the topic modeling, we implemented a text preprocessing pipeline. Each post was first converted to lowercase, and all nonalphabetic characters, redundant whitespace, and platform-specific artifacts (“amp,” “com,” “www,” “https”) were removed. URLs and hashtags were stripped. Tokenization and lemmatization were performed using the English-language SpaCy model (en_core_web_sm). During this process, only alphabetic tokens were retained, and standard English stop words, as defined by the Natural Language Toolkit corpus, were excluded.

To determine the most effective method for topic extraction, we systematically compared three topic modeling algorithms:

1. BERTopic: A neural topic modeling approach that leverages transformer-based document embeddings, density-based clustering, and class-based Term Frequency–Inverse Document Frequency (TF-IDF) to generate coherent topic representations [54].
2. LDA: A probabilistic generative model that assumes that documents are mixtures of latent topics, where each topic is characterized by a distribution over words and each document is represented as a distribution over topics [55].
3. Non-negative matrix factorization (NMF): A matrix decomposition method that factorizes the document-term matrix into 2 non-negative matrices representing topic-word and document-topic associations [56].

Each algorithm was applied separately to the dataset using its default parameters. For LDA and NMF, the preprocessed text was vectorized using CountVectorizer class from the scikit-learn library, and the vocabulary was restricted to the 1000 terms with the highest document frequency across the corpus. The resulting document-term matrix was weighted using TF-IDF. For these models, we varied the number of topics from 2 to 70 and computed the average coherence score (c_v) [57] to evaluate the topic quality. This metric assesses the semantic similarity among the top terms within each topic, providing an estimate of the conceptual coherence. In contrast, BERTopic automatically determined the number of topics using density-based clustering.

For each social media platform and hashtag subset, we selected the algorithm and number of topics that yielded the highest coherence score. This ensured that both the modeling approach and the number of topics reflected the most coherent and meaningful segmentation of the data. To label the topics, we extracted the top 15 most representative terms based on their TF-IDF weights from the topic–word matrix. These key terms, in combination with representative post examples, informed the assignment of descriptive topic labels.

### Misinformation Analysis

**Figure 1.**
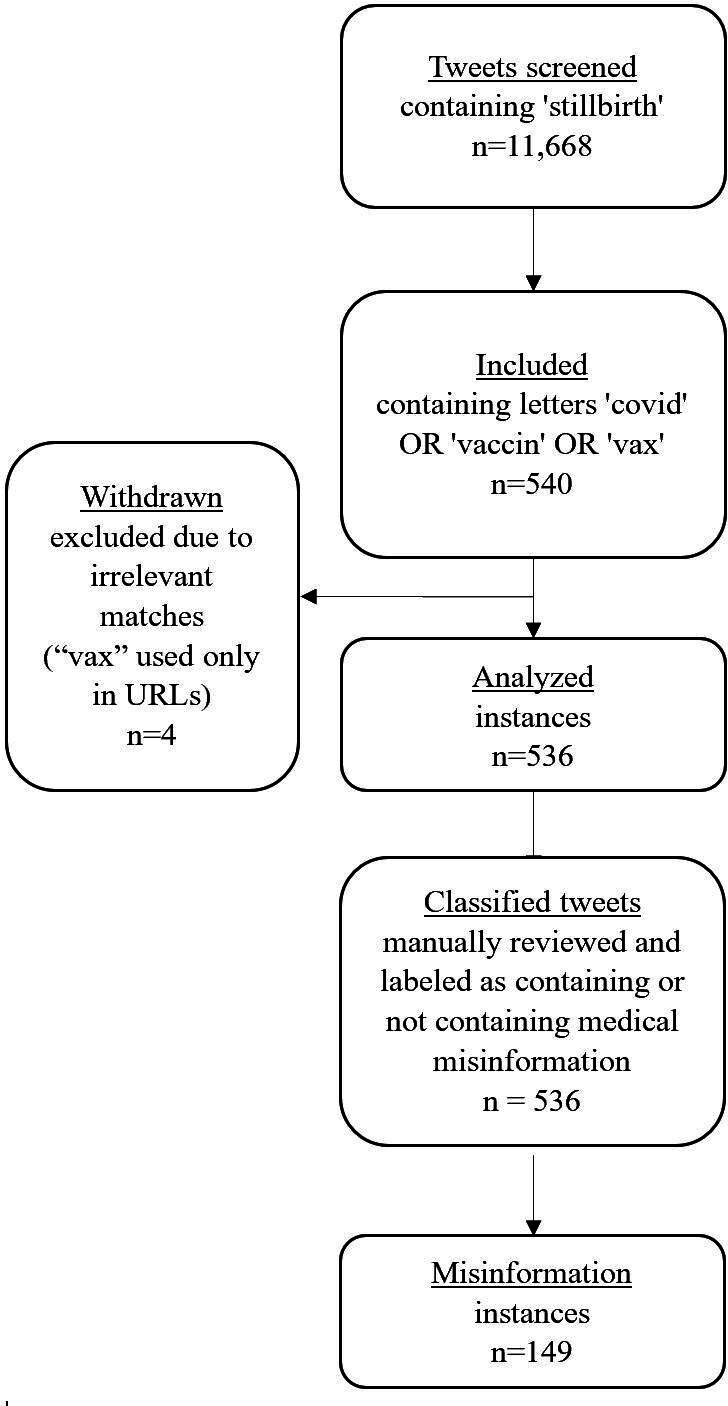
Flowchart of source data generation regarding misinformation on COVID-19 vaccination and stillbirth (2020-2024).

To identify and assess the prevalence of posts, including misinformation on COVID-19 vaccination and stillbirth, we screened all tweets on X. Instagram was not part of this analysis due to its shorter data collection window and the absence of relevant cases containing medical misinformation on this topic during the sampled period. The detailed screening process is illustrated in Figure 1 and will be further elaborated in the “Results” section of the misinformation analysis. Tweets were initially screened based on the presence of the term “stillbirth.” From this set, tweets relating to vaccination or COVID-19 were selected by identifying any variation of the terms “covid,” “vaccin,” or “vax” (“vaccin” was used as a stem to capture variations such as vaccine, vaccines, vaccination, and vaccinated, while “vax” is a common informal abbreviation of “vaccine” [58]). Tweets containing these terms solely in irrelevant contexts, such as within URLs, were manually withdrawn. All remaining tweets were manually reviewed by a medical expert specializing in stillbirth and maternal health, with over 10 years of research experience in pregnant women and more than 6 years of clinical practice and responsibility for research and development in the Department of Obstetrics and Gynecology at a hospital in Israel.

Misinformation screening was informed by established misinformation detection frameworks, including World Health Organization (WHO) Guidelines [59] and peer-reviewed scientific evidence [44-46]. Misinformation was defined as content containing claims that contradicted the WHO’s guidelines and peer-reviewed scientific evidence, or that cited noncredible or disproven sources. Ambiguous cases were resolved through discussion with a second reviewer until consensus was reached. Examples of tweets classified as misinformation are provided in the “Results” section.

### Sentiment and Emotion Analyses

#### Overview

For each post, we analyzed both the emotions and sentiments expressed in the text. Emotion analysis identifies specific feelings, such as sadness and joy, while sentiment analysis categorizes the overall tone of a sample as positive, negative, or neutral. This was undertaken to understand the range of emotions associated with stillbirths across Instagram and X.

#### Sentiment Analysis

The sentiment analysis (positive, negative, and neutral) was performed using Twitter-roBERTa-base for Sentiment Analysis [60], a RoBERTa-base model that was pretrained on approximately 124 million tweets collected between January 2018 and December 2021. The model was fine-tuned specifically for sentiment analysis using the TweetEval benchmark, ensuring high accuracy and relevance for analyzing social media text.

#### Emotion Analysis

The emotion analysis (anger, disgust, fear, joy, neutrality, sadness, and surprise) was conducted using the Emotion English DistilRoBERTa-base model [61]. This model is a fine-tuned checkpoint of DistilRoBERTa-base, designed to classify emotions in English text data. The model was trained on 6 diverse datasets, consisting of a balanced subset of nearly 20,000 observations (2811 observations per emotion). Of this subset, 80% (16,000/20,000) was used for training and 20% (4000/20,000) for evaluation, achieving an evaluation accuracy of 0.66.

To enable a fair comparison between Instagram and X in both sentiment and emotion analysis, we restricted the cross-platform analysis to posts collected during the overlapping time frame of 2023‐2024. For each platform, we calculated CIs for the proportion of posts that were classified under each sentiment and emotion category using Wilson score CIs [62]. CIs were computed at the 95% confidence interval. The Wilson method was chosen for its ability to provide more reliable estimates of the true underlying proportions, as it accounts for both the sample size and observed proportion [63].

In addition, since the dataset from X covered a broader date range (2020‐2024), sentiment and emotion scores are presented as aggregated by year, allowing for the identification of temporal trends in public emotional responses.

### Image Analysis

#### Overview

#### Sample Size Calculation

To determine the required sample size for statistical representativeness, we applied the standard sample size formula for finite populations [64]. The calculation was based on a total population of Instagram posts tagged with #stillbirth, using a 95% CI and a ±5% margin of error.

#### Random Sampling

To support both category development and classification reliability, 3 distinct random samples of images were drawn from the full dataset. The sample size for each was based on the calculation described in phase 1, ensuring statistical representativeness. The first sample was used to develop the category codebook, while the remaining 2 were independently analyzed for image classification. Using 2 classification samples enabled us to assess the stability of category distributions across different subsets and reduce the risk of sampling or selection bias.

#### Development of the Category Codebook

The first sample from phase 2 of the initial Instagram images was manually open-coded, allowing for recurring visual motifs and semantic themes. Overlapping codes were then merged, and discrepancies were resolved through discussion among the research team. This iterative process led to the creation of a structured codebook consisting of 8 specific categories, organized under 4 overarching themes: graphic expressions, parenthood, remembrance, and others.

Table 1 presents the finalized codebook, outlining the main categories, subcategories, operational definitions, and representative examples that guided the classification of Instagram images tagged with #stillbirth.

**Table 1.** Codebook for visual content categories in stillbirth-related Instagram posts.

Main category	Subcategory	Description	Examples
Graphic expressions	Image of text	Images in which the primary content is written text	Quotes, facts, and supportive statements
Graphic expressions	Illustrations	Artistic visuals created either digitally or by hand	Drawings, paintings, and graphic designs
Parenthood	Family moments	Personal images depicting shared family interactions, celebrations, or milestones	Family gatherings, holiday celebrations, birthday parties, siblings, and “rainbow babies” (infants born after a stillbirth)
Parenthood	Motherhood	Images depicting a mother or woman	Selfies of the mother, mother holding a baby
Remembrance	Stillborn infant	Photographs of stillborn babies	The baby in a hospital setting, being held by parents, partial views (eg, head, hand, and foot)
Remembrance	Memorial tributes	Images created to honor and remember the baby	Gravesites, remembrance candles, memory boxes, and baby footprint
Remembrance	Pregnancy	Visuals honoring a pregnancy that ended in loss	Ultrasound images, pregnancy tests, and gestational images
Other	None	Images unrelated to the core thematic categories	Food, fashion, fitness, or general lifestyle content

#### Annotator Training and Manual Classification

Two independent annotators (research assistants) were involved in the manual classification process. They participated in a 1-hour calibration session using the images used in the development of the codebook. During this session, ambiguous cases were reviewed, and coding definitions were refined to ensure consistent interpretation. Following training, both annotators independently classified each image in the 2 study samples using the codebook. Disagreements were resolved through collaborative discussion; when a consensus could not be reached, a third reviewer adjudicated the final label.

#### Interrater Reliability Assessment

The interrater reliability between annotators was assessed using Cohen Kappa statistic to evaluate the level of agreement beyond chance [65]. To estimate the uncertainty around the Kappa value, a nonparametric bootstrap method with 1000 resamples was applied [66]. The resulting distribution was used to derive a 95% CI for the Kappa estimate.

#### Cross-Sample Consistency Check

To verify that the 2 independent image classification runs yielded statistically comparable category distributions, a Pearson correlation was computed between the raw category counts from each sample [67]. In addition, a chi-square goodness-of-fit test was conducted to assess whether the observed frequencies deviated significantly from the expected values [68].

### Ethical Considerations

The data collection process and analysis were approved by the Emek Yezreel College Ethical Review Board (approval number 2024‐136 YVC EMEK). As the research relied solely on publicly available social media data and did not involve direct interactions with individuals, informed consent was not applicable. No compensation was offered or provided, as the study did not involve the direct participation of human participants. All data used in the analysis were publicly available and did not contain personally identifiable information.

## Results

### Data Collection

On X, we collected posts containing the hashtag #stillbirth, while on Instagram, we retrieved posts tagged with both #stillbirth and #stillbirthawareness. A total of 27,395 posts were collected across platforms based on the volume of data that was accessible via the respective APIs, including 7,415 Instagram posts with the hashtag #stillbirth (2023–2024), 8,312 Instagram posts with the hashtag #stillbirthawareness (2023–2024), and 11,668 posts on X with the hashtag #stillbirth (2020–2024).

Multimedia Appendix 1 displays the annual distribution of posts retrieved from Instagram and X. Due to API limitations, the Instagram dataset primarily covers the 2 years preceding data collection (2023‐2024). Therefore, a longitudinal analysis was not feasible for Instagram. In contrast, the X dataset spans a broader period with a relatively consistent volume of tweets from 2020 to 2024, enabling temporal analysis.

### Hashtag Co-Occurrence Networks

We constructed 2 separate hashtag co-occurrence networks based on the datasets from the years 2023‐2024. The first network, derived from Instagram posts containing #stillbirth, consists of 10,936 unique hashtags (nodes) and 225,912 connections (edges). The second network, built from tweets on X that contain #stillbirth, contains 3854 unique hashtags and 22,295 edges.

Multimedia Appendix 2 provides a complete list of all hashtags from both X and Instagram, along with their corresponding PageRank scores. The primary hashtag (#stillbirth) that was used to collect the data was excluded from the ranking, as expected, since it was central to the dataset. Across both platforms, hashtags, such as #miscarriage, #pregnancyloss, #babyloss, #infantloss, and #grief consistently appear at the top, reinforcing their strong connection to stillbirth-related discussions.

The hashtags can be categorized into distinct themes:

1. Loss-related hashtags: These directly reference different types of pregnancy and infant loss, including #pregnancyloss, #pregnancyandinfantloss, #babyloss, #tfmr (termination for medical reasons), and #infantloss.
2. Emotional expression hashtags: The tags, such as #grief and #stillbornstillloved, convey personal emotions and mourning.
3. Awareness-related hashtags: These highlight efforts to raise awareness about stillbirth and related issues, including #babylossawareness, #neonataldeath, #pregnancylossawareness, #miscarriageawareness, and #infantlossawareness.
4. Support-related hashtags: These hashtags are used to foster community support and connection, and they include #babylosscommunity, #griefsupport, #miscarriagesupport, #stillbirthsupport, and #babylosssupport.
5. Encouragement-related hashtags: These provide emotional encouragement and acknowledgment for bereaved families, including hashtags, such as #bereavedparents, #bereavedmother, and #rainbowbaby (which refers to a live baby being born after a pregnancy loss, symbolizing hope after grief).
6. Health-related hashtags: Among the top 30 hashtags on X, several are linked to medical topics, including #COVID19. A further examination of tweets relating to COVID-19 is described in the misinformation analysis section.

**Figure 2.**
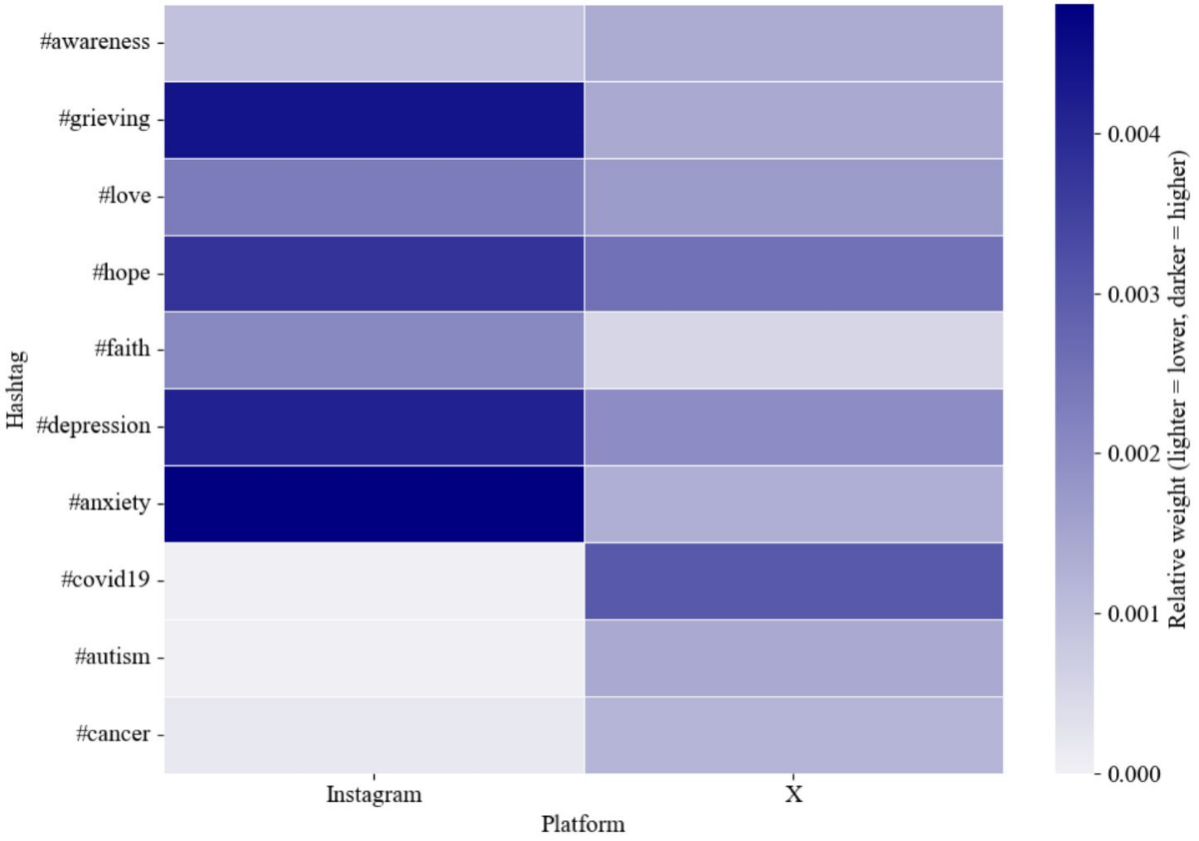
Comparison of selected hashtags across Instagram and X (2023‐2024) based on their relative weights.

Figure 2 presents a comparison of selected hashtags relating to emotional and health themes across Instagram and X, based on their relative weights within each platform’s hashtag co-occurrence network. Multimedia Appendix 2 also provides the full list of hashtags and their corresponding relative weights. Darker shades in the figure indicate a higher relative prominence of a hashtag on the given platform. The results show that hashtags such as #anxiety, #depression, and #grieving appear with greater relative weight on Instagram, suggesting a stronger emphasis on emotional expression and mental health within that platform. In contrast, hashtags such as #COVID19, #autism, and #cancer are relatively more prominent on X, possibly reflecting more informational or public health-oriented discourse. Hashtags such as #hope, #love, and #faith exhibited slightly higher relative weights on Instagram than on X.

**Figure 3.**
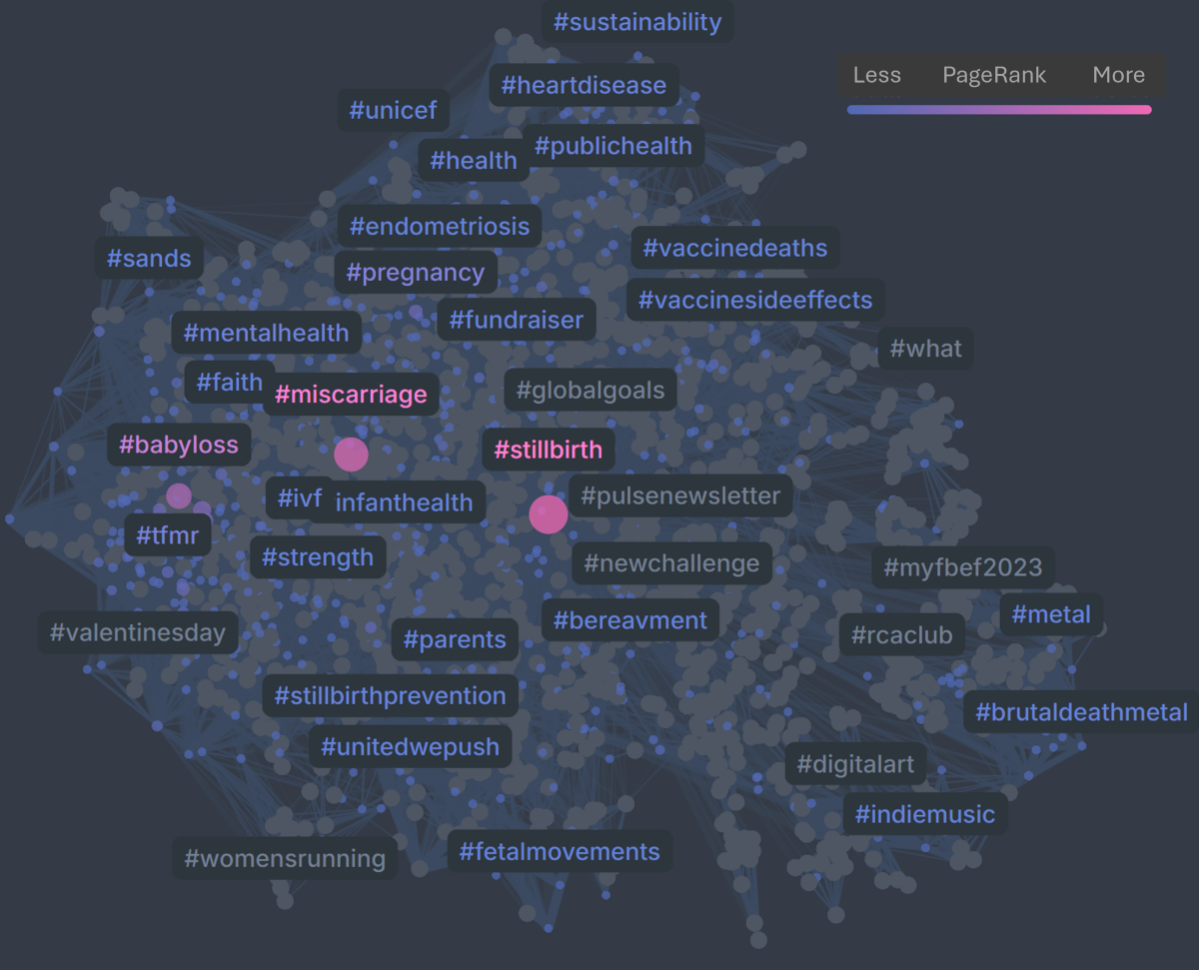
Hashtag co-occurrence network for #stillbirth on X (2023‐2024).

**Figure 4.**
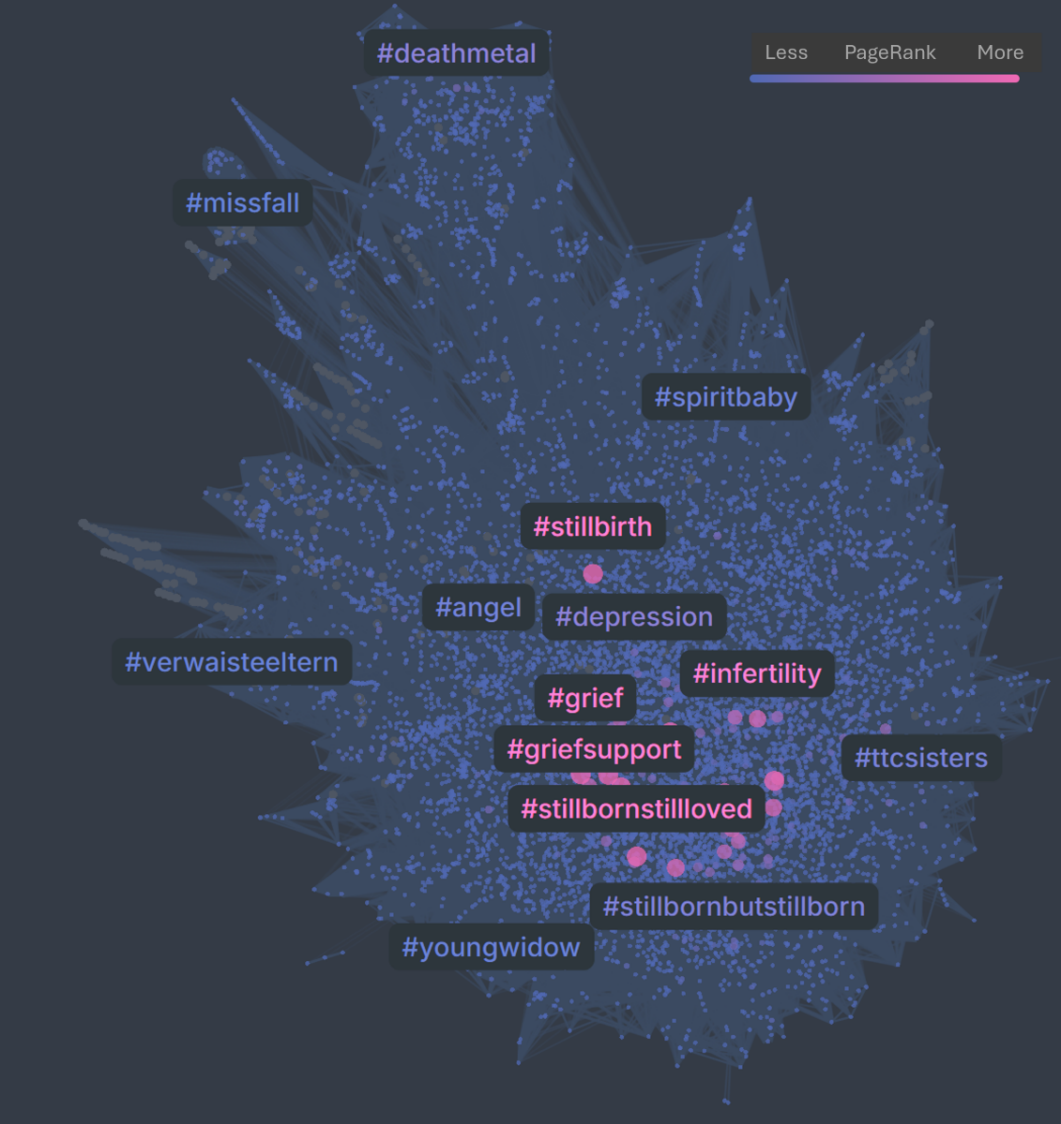
Hashtag co-occurrence network for #stillbirth on Instagram (2023‐2024).

Figures3  4 present the hashtag co-occurrence networks for **#**stillbirth on X and Instagram, respectively, for the period 2023‐2024. In both networks, nodes represent hashtags, with node size, node color, and hashtag label color corresponding to PageRank scores, and edges weighted by the frequency of hashtag co-occurrence. The X network (Figure 3) positions **#**stillbirth within a broader and more heterogeneous structure, linking to bereavement-related hashtags (eg, #miscarriage, #babyloss, and #mentalhealth) alongside peripheral connections to broader health topics, such as vaccinations. In contrast, the Instagram network (Figure 4) forms a denser and more cohesive cluster centered on emotional support and bereavement, reflecting a concentrated community focus on personal loss.

### Topic Modeling

To identify the dominant narratives surrounding stillbirth on social media, we applied and evaluated 3 topic modeling algorithms, such as LDA, NMF, and BERTopic. Table 2 presents the top 3 configurations for each dataset—X posts tagged with #stillbirth, Instagram posts tagged with #stillbirth, and Instagram posts tagged with #stillbirthawareness—ranked by their coherence scores. These results highlight the most effective combinations of algorithm and topic count for capturing coherent themes. Multimedia Appendix 3 presents the full results of the topic modeling evaluation process, conducted across the 3 algorithms, for each social media platform and hashtag.

**Table 2.** Top 3 topic modeling results per hashtag and platform, sorted by their coherence scores; the selected result for each hashtag–platform combination is highlighted in bold.

Social media	Hashtag	Algorithm	Number of topics	Coherence score
Instagram	#stillbirthawareness	BERTopic	68	0.639
Instagram	#stillbirthawareness	BERTopic after reducing outliers	68	0.602
Instagram	#stillbirthawareness	NMF^a^	3	0.629^b^
Instagram	#stillbirthawareness	NMF	4	0.624
Instagram	#stillbirth	NMF	4	0.846^b^
Instagram	#stillbirth	NMF	5	0.821
Instagram	#stillbirth	NMF	3	0.821
X	#stillbirth	NMF	4	0.816^b^
X	#stillbirth	NMF	3	0.813
X	#stillbirth	BERTopic	105	0.813

a NMF: Non-negative matrix factorization.

b Selected result for each hashtag–platform combination.

In Instagram posts tagged with #stillbirthawareness, the BERTopic model initially achieved the highest coherence score (0.639), with 68 topics. However, the model classified 4230/8312 posts as posts that could not be assigned to any topic, indicating a substantial proportion of outliers that were not grouped into meaningful clusters. This suggests that despite its high coherence, the model may have struggled to capture thematic consistency across a large portion of the dataset. To address this, we applied BERTopic’s built-in outlier reduction method, which reassigns unclustered posts to the most semantically similar existing topics based on their embeddings. This adjustment reduced the number of outliers to only 10, thereby improving the topic coverage and interpretability. However, this came at the cost of a lower coherence score (0.602). Given this trade-off, we selected the configuration that achieved the highest coherence score, which was produced by NMF with 3 topics (coherence=0.629), for the final analysis.

For the Instagram dataset tagged with #stillbirth, the best results were obtained using the NMF algorithm with 4 topics, yielding a coherence score of 0.846. Similarly, for the X dataset tagged with #stillbirth, the highest coherence score (0.816) was also achieved using the NMF algorithm configured with 4 topics.

Table 3 presents the topics that were extracted using the topic modeling of social media posts across Instagram and X containing the hashtags #stillbirth and #stillbirthawareness. For each topic, the topic name and number of associated posts are provided.

**Table 3.** Topics identified through topic modeling for #stillbirthawareness and #stillbirth on Instagram and #stillbirth on X, including topic name and count.

Social media platform	Hashtag	Topic number	Topic name	Post count
X	#stillbirth	1	Awareness and remembrance	5655
X	#stillbirth	2	Support	2946
X	#stillbirth	3	Risks, prevention, and research	2390
X	#stillbirth	4	Inspiring stories	677
Instagram	#stillbirth	1	Remembering and honoring lost babies	3989
Instagram	#stillbirth	2	Support	2734
Instagram	#stillbirth	3	Natural remedies and healing	225
Instagram	#stillbirthawareness	1	Remembering and honoring lost babies	4315
Instagram	#stillbirthawareness	2	Awareness and remembrance	1590
Instagram	#stillbirthawareness	3	Support and prevention	2407

For X posts with the hashtag #stillbirth, the most dominant topic, with 5655 posts, was “Awareness and remembrance,” reflecting deeply emotional language around loss, love, and awareness of stillbirth experiences. The second most common theme, “Support” (2946 posts), centered on expressions of gratitude and requests for help, emphasizing a need for practical and emotional support. The third topic, “Risks, prevention, and research”, included 2390 posts and focused on calls to action, policy discussions, and education about prevention. Finally, “Inspiring stories”, although smaller in volume (677 posts), captured narratives of personal stories, faith, and resilience.

For Instagram posts under the hashtag #stillbirth, we excluded 1 topic containing 467 posts in different languages from the table. “Remembering and honoring lost babies” (3989 posts) was the most prevalent topic, emphasizing sentiments of love and grief and often referring to significant dates, such as birthdays or anniversaries of the baby’s death. The second topic of support (2734 posts) consisted mostly of texts providing guidance and assistance for families coping with stillbirth. Many of these posts focused on offering emotional support, sharing helpful resources, and raising awareness about available services for grieving parents. A smaller topic, “Natural remedies and healing” (225 posts), featured advertisements for natural products and alternative healing methods.

For #stillbirthawareness on Instagram, the theme of “Remembering and honoring lost babies” was the dominant topic (4315 posts), reflecting personal expressions of grief, love, and remembrance. The “Awareness and remembrance” category (1590 posts) contained discussions surrounding stillbirth awareness and remembrance initiatives, with notable mentions of October, which is internationally recognized as Pregnancy and Infant Loss Awareness Month. The “Support and prevention” category (2407 posts) focused on providing help and guidance for families, advocating for stillbirth prevention efforts, and sharing information from organizations.

### Misinformation Analysis

As shown in Figure 1, out of the tweets containing the term “stillbirth” and additional references to *“*covid*,” “*vaccin*,”* or *“*vax*,”* a total of 536 tweets were manually analyzed. Following classification, 27.8% (149/536) were identified as containing medical misinformation relating to stillbirth and vaccination.

These tweets frequently included unsubstantiated claims suggesting causal links between COVID-19 vaccination and adverse pregnancy outcomes, such as miscarriage or stillbirth. Many relied on anecdotal evidence that was presented through unofficial websites or films, expressed distrust in public health authorities, and amplified conspiracy theories portraying vaccines as harmful, poisonous, or intentionally designed to cause widespread harm or even genocide.

Examples include the following:

1. “i wouldnt be taking vaccine look up #stillbirth”
2. “#vaccineinjuries #vaccinegenocide #vaers #vaccine #vaccines #suddendeaths #fertility #miscarriage #suddendeath #diedsuddenly #stillbirth #depopulationagenda #depopulation #fda”
3. “Why are there so many stillbirths and pregnancy issues with vaccinated women? #mrna #ace2 #stillbirth #miscarriage #phizer #vaccineinjuries #crimesagainsthumanity”
4. “Just watched a film !!! I knew the #vaccines weren’t safe, but to see all the embalmers describing the clots, the #stillbirth data, and the military #whistleblowers was just shocking! ”
5. “Please stop telling #women to take these #vaccines, it’s killing their #babies!!! If you can’t do that then stop practicing medicine!! #miscarriages #stillbirth #vaers”

### Sentiment and Emotion Analyses

**Figure 5.**
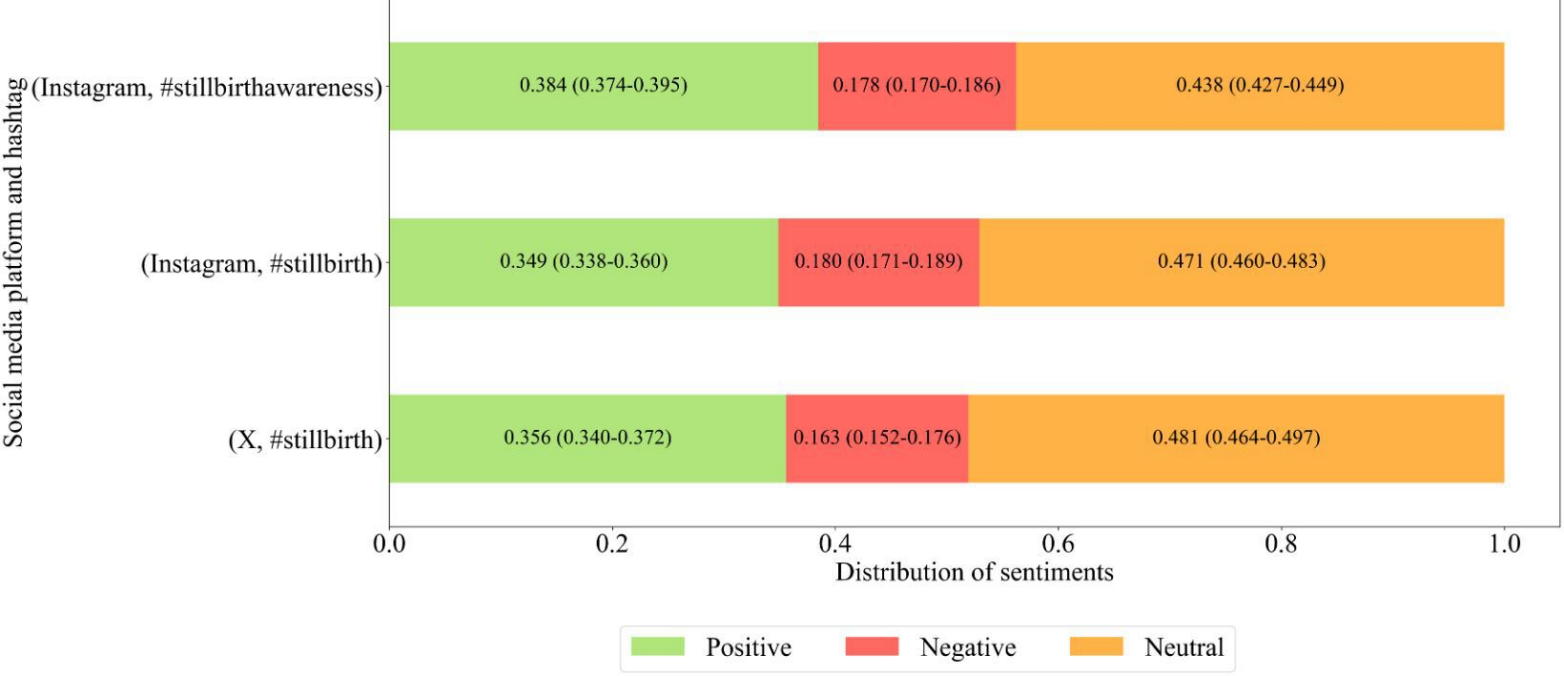
Sentiment distribution (positive, negative, and neutral) across Instagram posts with the hashtags #stillbirth and #stillbirthawareness and X posts with the hashtag #stillbirth (2023-2024). Values represent the mean observed proportion of posts in each sentiment category, with Wilson 95% CIs.

Figure 5 presents the average sentiment distribution of positive, negative, and neutral sentiments. For Instagram, the hashtags analyzed were #stillbirth and #stillbirthawareness, and for X, the hashtag analyzed was #stillbirth. The same time period, 2023‐2024, was analyzed for both social media platforms. When comparing the 2 hashtags on Instagram, #stillbirthawareness had a higher proportion of positive sentiments and a lower proportion of negative sentiments than #stillbirth. In addition, when examining the same hashtag of #stillbirth across Instagram and X, the proportion of positive sentiments on Instagram was higher, while that of negative sentiments was lower.

**Figure 6.**
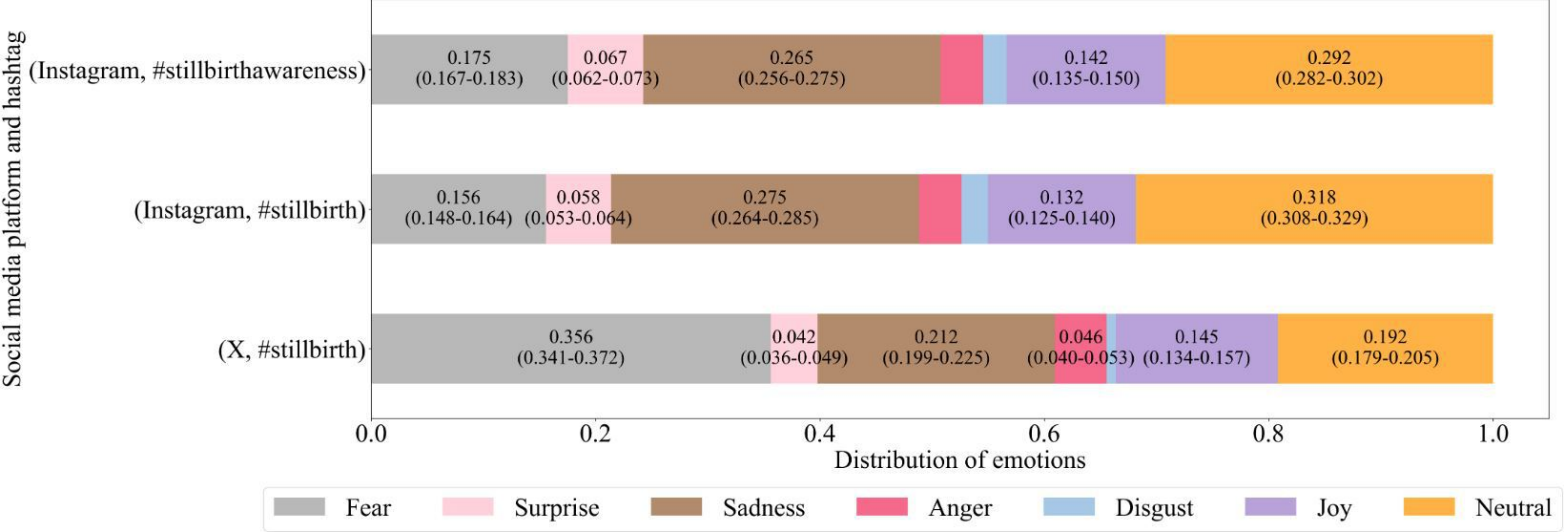
Distribution of emotions (fear, surprise, sadness, anger, disgust, joy, and neutrality) across Instagram posts with the hashtags #stillbirth and #stillbirthawareness and X posts with the hashtag #stillbirth (2023–2024). Values represent the mean observed proportion of posts in each emotion category, with Wilson 95% confidence intervals.

Figure 6 shows the distribution of emotions—fear, surprise, sadness, anger, disgust, joy, and neutrality—for the same set of hashtags across the 2 platforms. Emotions with scores below 0.04 were excluded from the figure annotations to enhance the figure clarity. Three dominant emotions were consistently observed across all hashtags on both X and Instagram: neutrality, sadness, and fear. In contrast, surprise, anger, and disgust were less commonly expressed on both platforms. When comparing the 2, fear was more pronounced on X, whereas sadness and joy appeared more frequently on Instagram.

**Figure 7.**
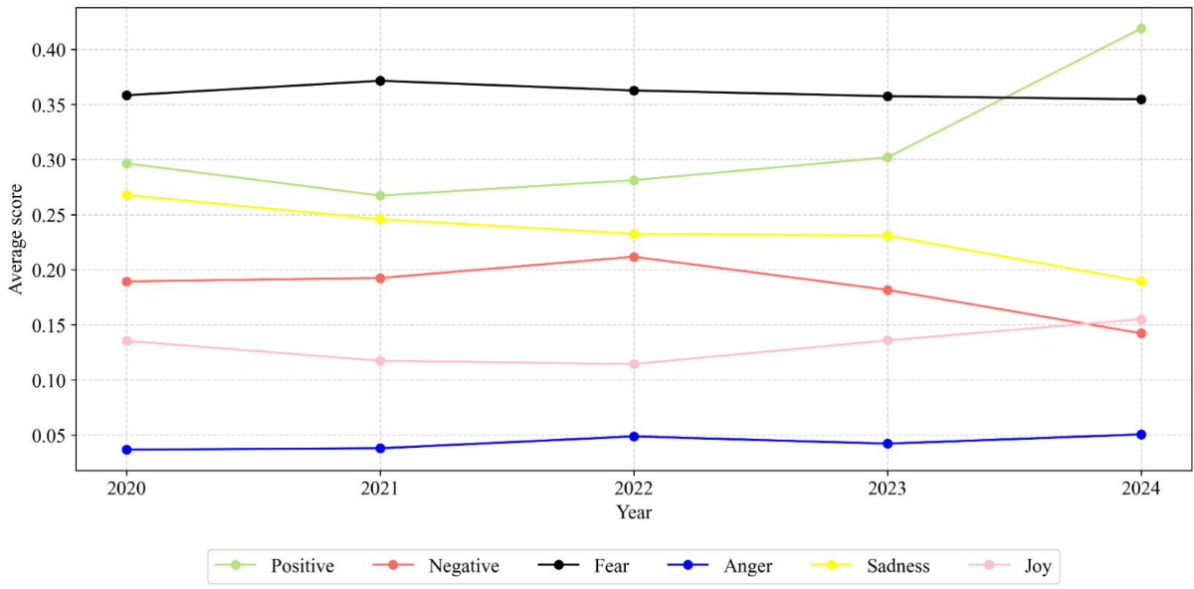
Annual trends in positive, negative, fear, anger, sadness, and joy scores for #stillbirth on X (2020-2024).

Figure 7 presents the average annual scores for sentiments (positive and negative) and selected emotions (fear, anger, sadness, and joy) in posts containing the hashtag #stillbirth on X between 2020 and 2024. The most prominent emotion across the entire period was fear, which consistently registered the highest among all emotional categories—particularly notable in the early years of the COVID-19 pandemic (2020‐2021). Positive sentiments demonstrated a gradual rise throughout the period, with a sharp increase observed in 2024. In contrast, negative sentiments increased until 2022, after which they declined. Sadness steadily decreased over time while joy increased modestly during 2023‐2024. Anger exhibited minimal year-to-year variation, remaining consistently low.

### Image Analysis

To ensure statistical representativeness, the required sample size was calculated based on a population of n=7415 Instagram images, a 95% CI, and a ±5% margin of error, resulting in a target sample of 366/7415. The first sample of 366 images was used to develop the codebook. Two additional independent samples of 366 images each were subsequently categorized by 2 trained annotators.

The interrater reliability for the 2 coding samples was assessed using Cohen Kappa statistic, which measures agreement beyond chance and is reported with its 95% CI. In both samples, the agreement was strong: in sample 1, κ=0.837 (mean 0.836, SD 0.03; 95% CI 0.773‐0.891), while in sample 2, κ=0.821 (mean 0.821, SD 0.03; 95% CI 0.755‐0.879). The overlapping CIs indicate that there was no substantial difference in agreement between the 2 assessments, supporting the consistency and reliability of the coding procedure.

After calculating the distribution of categories within each sample, their similarity was assessed. The results demonstrated a near-perfect correlation between the 2 distributions based on their Pearson correlation (*r*=0.999; *P*<.001), along with a nonsignificant difference in categorical frequencies (*χ*²_7_=12.4; *P*=.09). These findings support the decision to pool the 2 samples for subsequent analyses, based on both the high interrater agreement and the absence of statistically significant divergence between the category distributions.

Table 4 presents the distribution of images across topics for the 2 independent samples (n=366 each) based on the predefined codebook. In both samples, the dominant category was “Image of text,” comprising text-based graphics with uplifting quotes, awareness messages, and supportive content relating to stillbirth, shared by both individuals and organizations. Excluding this category, most images were personal and intimate, with remembrance being the most common theme (44/366 in the first sample and 38/366 in the second). These included depictions of stillborn infants, memorial tributes, and symbolic representations of loss.

**Table 4.** The categorization of the randomly selected Instagram images, using the hashtag #stillbirth, into 4 themes: graphic expressions*,* parenthood*,* remembrance, and other (n=366).

Theme	Topic	Distribution sample 1, n (%)	Distribution sample 2, n (%)
Graphic expressions	Image of text	263 (71.9)	277 (75.7)
Graphic expressions	Illustrations	10 (2.7)	9 (2.5)
Parenthood	Family moments	11 (3)	5 (1.4)
Parenthood	Motherhood	5 (1.4)	10 (2.7)
Remembrance	Stillborn infant	14 (3.8)	14 (3.8)
Remembrance	Memorial tributes	25 (6.8)	22 (6)
Remembrance	Pregnancy	5 (1.4)	2 (0.5)
Other	None	33 (9)	27 (7.4)

## Discussion

### Principal Findings and Comparison With Existing Literature

The results of the hashtag co-occurrence network analysis for #stillbirth during 2023‐2024 (Figures2^4^) indicated that Instagram posts emphasized emotional expression and mental health, whereas the discourse on X was more oriented toward informational and public health-related content. Interestingly, X showed a strong presence of health-related hashtags, with #COVID19 ranking among the most prominent.

Analyzing tweets relating to vaccination or COVID-19 (Figure 1) revealed that in nearly 27.8% (149/536) of cases, the information that was shared was inaccurate. These findings indicate that stillbirth-related discourse on X is not solely focused on emotional and community support but also intersects with broader health debates and misinformation narratives.

Recent studies have explored machine learning approaches to detect health misinformation on social media [41,42,69,70,71]; however, gaps remain in real-time monitoring and addressing emotionally charged topics, such as stillbirth. The prevalence of inaccurate information that we identified underscores the need for targeted interventions to correct misinformation, particularly in sensitive health contexts. Of note, the immediate and widespread reach of platforms, such as X, means that information, both accurate and inaccurate, spreads quickly. Engaging authoritative figures is critical to steer the narrative [70]. Our findings highlight the spread of misinformation within social media and emphasize the importance of ensuring that accurate, evidence-based information reaches the public. It is essential to combat misinformation, particularly for individuals who have experienced stillbirth and may struggle with self-blame, fearing that vaccinations contributed to their loss. Providing clear, evidence-based information on social media can help to dispel these concerns, alleviate unnecessary guilt, and support informed decision-making regarding maternal health.

The evaluation of 3 topic modeling algorithms (NMF, LDA, and BERTopic) identified NMF as the most suitable method for processing our data (Table 2), based on its consistently superior coherence scores. This finding aligns with prior research demonstrating that NMF often outperforms traditional models, such as LDA in terms of topic coherence, particularly when applied to the short and noisy texts that are commonly found in social media contexts [72-74]. LDA is known to exhibit topic overlap and reduced stability when handling sparse data. Although BERTopic introduces an innovative approach by incorporating transformer-based embeddings, it frequently yields a high proportion of outliers, which undermines the interpretability [75]. Given the informal and fragmented nature of our datasets, NMF provided the most coherent and interpretable topic structures, making it the preferred algorithm for our analysis.

Our topic modeling analysis of posts on both X and Instagram shows that users predominantly share personal experiences of stillbirth, with dominant themes of honoring and remembering their babies, marking milestones (birthdays and anniversaries), and raising awareness and support. These findings are consistent with prior research on miscarriage and pregnancy-loss discourse in social media. For example, Dubbelman et al [76] and Putri et al [77] highlight themes of grieving, healing, support, gratitude, and informational posts, which reinforces our observation of remembrance and awareness-raising as central to stillbirth discourse. Similarly, Mercier et al [30] identified emotional disclosure as central to miscarriage posts on Instagram, a pattern that aligns with the personal storytelling we observed, though our findings extend these works by showing how these disclosures are accompanied by highly visual memorial practices unique to stillbirth. Callen and Oxlad [31] documented 5 forms of social support (informational, emotional, esteem-based, tangible, and network-based) in a Facebook miscarriage group, which parallels the supportive interactions present in our dataset and thus strengthens the interpretation of social media as a support infrastructure.

Our results extend prior work by showing that visual memorialization is a distinctive feature of stillbirth discourse, consistent with continuing bonds theory [21]. Although text-based images appear numerically common in our dataset (Table 4), stillbirth posts frequently incorporate highly expressive commemorations—such as photographs of stillborn infants, memorial tributes, and symbolic imagery—that differ from disclosures in miscarriage studies [76,77]. Unlike miscarriage, stillbirth typically involves full-term labor [1] and the physical birth of a visibly developed baby, intensifying the emotional impact and need for remembrance. Consequently, stillbirth-related content on Instagram not only displays unique platform dynamics and emotional depth but also illustrates how parents maintain continuing bonds with their infants through visual tributes. These images serve as enduring memorials that affirm the baby’s existence, validate parental grief, and resist societal pressures to minimize or hasten recovery.

This visually anchored pattern aligns with research on stillbirth in social media. Hayman et al [78] showed that parents use photographs, symbolic imagery, linking objects, and rituals on Facebook to maintain ongoing relationships with the “born still,” extending continuing bonds theory [21] into shared social spaces. Similarly, Sani et al [34] interpreted YouTube videos by bereaved mothers as modern mortuary rituals, transforming the platform into a digital cemetery. Within the broader literature on child death, not limited to stillbirth, Keskinen et al [79] identified images of child portraits, tombstones, mementos, drawings, and postdeath photographs. These categories closely mirror those found in our Instagram dataset, suggesting cross-platform consistency in the symbolic repertoire of grief. The close alignment with studies of child bereavement underscores that parents experiencing stillbirth engage in practices of remembrance akin to those of parents grieving a child, exemplifying continuing bonds theory, where stillbirth is affirmed as the genuine loss of a child and parents sustain their connection through symbolic and visual forms of remembrance.

Our sentiment analysis revealed a higher proportion of positive than negative sentiments (Figure 5)—an unexpected pattern for stillbirth. Manual review revealed that “highly positive” posts on Instagram and X often mark milestones, remembrance, and love (eg, “Happy heavenly 14th birthday” and “Birthday cake made with love”), where positive words appear within grief. This exposes a limitation of automated sentiment classifiers, which may overascribe positivity in memorial contexts [80]. A continuing-bonds lens helps explain the mix, as users sustain ties to their babies through commemorations and affirmations [21,22], while platform communities provide support and alleviate disenfranchised grief [24]. The positive side of this mixed sentiment described above can be explained, at least in part, by incorporating continuing bonds theory into social media research. Such behavior can enable ongoing connections with posters’ memories of their babies [21,22]. This platform fosters community support and alleviates disenfranchised grievances [24].

Figure 6 shows that fear and sadness dominate over joy and neutral expressions, yielding an overall negative tone around stillbirth. Cross-platform differences are clear, with fear more prevalent on X, whereas sadness and joy are more common on Instagram. In addition, Instagram exhibits more positive and less negative sentiment than X. These patterns align with platform norms—X’s news- and debate-orientation tends to surface health-related fear and negativity [81], while Instagram’s visual, image-centric format fosters more emotionally expressive content [82-84].

Instagram’s visual environment can help legitimize parental grief and build supportive communities. Public health efforts could use curated awareness posts featuring family stories and commemorative content, brief influencer-led campaigns, and simple participatory prompts (eg, “light a candle for your baby”) with targeted hashtags to normalize bereavement and strengthen public understanding of stillbirth.

Future research should further explore how X’s real-time affordances amplify fear-based misinformation during crises, such as COVID-19, compared with Instagram’s visual curation. This will allow health policymakers to include such platforms as specific challenges in their fight against misinformation that falsely heightens pandemic-related anxiety in relation to stillbirth and related health concerns.

### Strengths and Limitations

This study addresses key gaps in the literature on stillbirth discourse, including common limitations, such as small sample sizes, a single-platform focus, and a lack of emotion, visual content, and misinformation analyses. Using a large-scale, multiplatform (Instagram and X) dataset and a multimethod approach—including topic modeling, sentiment and emotion analyses, hashtag network analysis, manual image classification, and misinformation detection—this research provides a comprehensive understanding of how stillbirth is represented and discussed on social media. Notably, it highlights the unique visual memorialization practices on Instagram and the prevalence of misinformation on X, positioning the study as a substantial contributor to digital health communication and grief-related research fields.

This study has several limitations that should be considered when interpreting the findings. First, social media posts only reflect the content that users choose to share publicly, which introduces a selection bias. These expressions may not capture the full emotional journey or the more private aspects of grief that are experienced by families affected by stillbirth. While this limits the completeness of the emotional representation, our analysis focuses specifically on publicly shared narratives, providing valuable insights into the discourse that individuals elect to make visible in the digital public sphere.

Second, the study lacked demographic information about content creators. We could not determine whether a post was authored by a woman, a man, or an organization, nor infer details, such as the poster’s age, cultural background, or personal experience with stillbirth. This limited our ability to analyze how this discourse might differ across demographic groups. Future work should explore ways to ethically incorporate user metadata or analyze verified support group discussions where roles and identities may be more clearly indicated.

Third, the time frame of the Instagram data (limited to 2023‐2024 due to API restrictions) constrained our ability to conduct a longitudinal analysis. As a result, we could not observe how stillbirth-related discourse evolved over a broader period. In contrast, the X data spanned the period of 2020‐2024, only allowing for a temporal analysis on that platform. To enable a meaningful comparison between the 2 platforms, we restricted the time frame for both datasets to the overlapping period of 2023‐2024. This allowed for consistent cross-platform analysis while minimizing temporal biases.

Fourth, our reliance on automated sentiment and emotion analysis tools introduced limitations in interpreting emotional tone. While these transformer-based models are valuable for large-scale analysis, they are not always fully accurate and may struggle to capture context-dependent nuances, including emotional ambivalence, sarcasm, or irony [80]. To mitigate this limitation, we manually reviewed a subset of posts that had been classified with extreme sentiment scores. In addition, we conducted a deeper analysis of the emotions expressed in the posts—such as sadness, fear, and anger—by using emotion detection techniques that can capture more nuanced emotional states beyond the basic positive, negative, and neutral sentiment categories. This combination allowed us to interpret the complex emotional landscape associated with stillbirth-related content more thoroughly.

### Future Directions

Future research should incorporate user metadata, where available, to better distinguish between personal narratives and institutional messaging, providing an understanding of how different entities engage in stillbirth-related discussions on social media. Future studies should also incorporate historical data to enable comprehensive time-series analyses.

### Conclusions

This study provides a comprehensive view of stillbirth-related discourse on Instagram and X, showing platform-specific patterns. Instagram posts emphasized emotional expression and mental health, whereas X posts focused more on public health and informational topics (eg, COVID-19). Topic modeling indicated that discussions centered on honoring babies, raising awareness, and providing support. Sentiment analysis showed more positive than negative sentiment overall, reflecting resilience, hope, and remembrance; however, fear and sadness were the dominant emotions. Fear was more prevalent on X, while Instagram displayed more sadness and joy, consistent with its more personal, visually expressive environment. Image analysis highlighted Instagram’s visual tributes—such as photographs, gravesites, and symbolic imagery—which function as digital memorials that validate parental grief and community support.

Fear on X likely relates to the rapid, real-time spread of content and the presence of COVID-19 vaccine-related claims. Notably, 27.8% (149/536) of posts referencing stillbirth and vaccination or COVID-19 contained misinformation, underscoring the need for evidence-based communication and careful interpretation of platform dynamics. This study underscores the role of misinformation in shaping social media discourse, particularly on X, where inaccurate claims about COVID-19 vaccines and stillbirths appear and amplify fear-based narratives. Pregnant women who are exposed to such misinformation may avoid vaccination due to unfounded fears, while bereaved mothers may internalize guilt, believing that vaccination contributed to their loss. Ensuring that accurate health information reaches the public is essential for countering such harmful effects.

Future work could develop platform-aware approaches to counter misinformation and support bereaved communities. On X, agencies could collaborate with the WHO and local maternal-health organizations to deploy real-time, evidence-based messages (eg, verified infographics) timed to peaks in misinformation, potentially amplified through influencers and health advocates with lived experience. Real-time monitoring could help surface emerging myths and inform rapid responses. On Instagram, health care and maternal-health organizations could design guided hashtag campaigns that pair personal narratives with accurate resources to normalize conversation about loss. Training programs in digital health communication could help clinicians engage compassionately and effectively across platforms.

As this study lacked user demographics, engagement metrics, and historical data from both platforms, future studies could incorporate longitudinal traces and richer metadata to improve generalizability and track trends over time. Further work could also examine how X’s real-time affordances amplify fear-based narratives during crises and evaluate which platform-tailored interventions are most effective at reducing misinformation and supporting bereaved families.

## Supplementary material

10.2196/73980 Multimedia Appendix 1 Annual numbers of posts collected from Instagram and X.

10.2196/73980 Multimedia Appendix 2 Hashtag PageRank scores and relative weights on Instagram and X (2023–2024).

10.2196/73980 Multimedia Appendix 3 Topic modeling evaluation results by algorithm and platform (latent Dirichlet allocation, non-negative matrix factorization, and BERTopic).

## Abbreviations

API: application programming interface
LDA: latent Dirichlet allocation
NMF: non-negative matrix factorization
TF-IDF: Term Frequency–Inverse Document Frequency
WHO: World Health Organization
