# Supplementary figures and images for: Stillbirth Discourse on Instagram and X (Formerly Twitter): Content Analysis
Source: JMIR Infodemiology. 2025 Sep 24;5:e73980. doi: 10.2196/73980 (PMC12466797; doi:10.2196/73980)

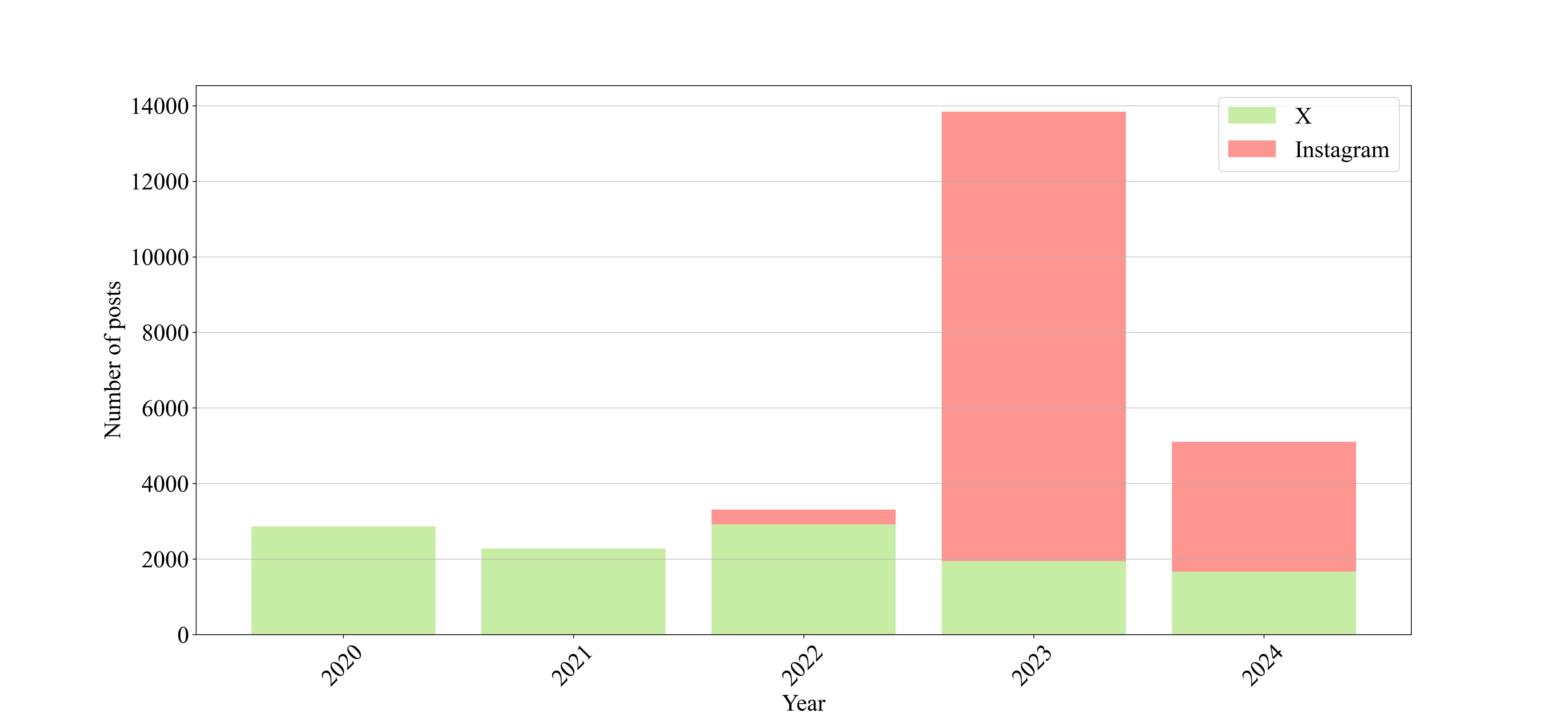

Supplement: Multimedia Appendix 1 [file infodemiology-v5-e73980-s001.jpg]
